# DNA Adjuvant Hydrogel‐Optimized Enzymatic Cascade Reaction for Tumor Chemodynamic‐Immunotherapy

**DOI:** 10.1002/advs.202308229

**Published:** 2024-01-15

**Authors:** Yan Zhao, Jiangnan Du, Zihui Xu, Lihua Wang, Lan Ma, Lele Sun

**Affiliations:** ^1^ Institute of Biomedical Health Technology and Engineering Shenzhen Bay Laboratory Shenzhen 518132 China; ^2^ Institute of Materiobiology Department of Chemistry College of Science Shanghai University Shanghai 200444 China; ^3^ Institute of Biopharmaceutical and Health Engineering Tsinghua Shenzhen International Graduate School Tsinghua University Shenzhen 518055 China; ^4^ Tsinghua‐Berkeley Shenzhen Institute Tsinghua University Shenzhen 518055 China; ^5^ State Key Laboratory of Chemical Oncogenomics Tsinghua Shenzhen International Graduate School Tsinghua University Shenzhen 518055 China

**Keywords:** chemodynamic therapy, DNA adjuvant hydrogel, enzyme cascade reaction, in situ vaccines, immunotherapy

## Abstract

Chemodynamic therapy (CDT) shows immense potential in cancer treatment as it not only directly kills tumor cells but also induces anti‐tumor immune responses. However, the efficacy of CDT is hampered by challenges in targeting CDT catalysts specifically to tumors using nanomaterials, along with the limitations of low H_2_O_2_ levels and short catalyst duration within the tumor microenvironment. In this study, DNA adjuvant hydrogel to arrange a glucose oxidase‐ferrocene cascade for continuously generating reactive oxygen species (ROS) from glucose in situ for tumor CDT combined with immunotherapy is employed. By precisely tuning the catalyst spacing with DNA double helix, ROS production efficiency is elevated by up to nine times compared to free catalysts, resulting in stronger immunogenetic cell death. Upon intratumoral injection, the DNA hydrogel system elicited potent anti‐tumor immune responses, thereby effectively inhibiting established tumors and rejecting re‐challenged tumors. This work offers a novel platform for integrated CDT and immunotherapy in cancer treatment.

## Graphical abstract

A DNA adjuvant hydrogel‐optimized enzyme cascade is developed for efficient chemodynamic therapy combined with immunotherapy, achieving the effective treatment of various solid tumor models on mice. The combination of immunoadjuvants continuously released from DNA hydrogels and tumor‐associated antigens released from immunogenic cell death of tumor cells can significantly inhibit tumor growth, recurrence, and metastasis.

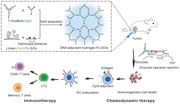

## 1 Introduction

Chemodynamic therapy (CDT) stands out as a promising approach for tumor treatment that employs iron‐based nanomaterials to catalyze endogenous H_2_O_2_ via Fenton or Fenton‐like reactions in an acidic tumor microenvironment (TME), resulting in the production of cytotoxic hydroxyl radicals (·OH) to destroy tumor cells.^[^ ^1^, ^2^, ^3^ ^]^ More importantly, the reactive oxygen species (ROS) generated during the CDT can effectively induce immunogenic cell death (ICD), leading to the exposure and release of damage‐associated molecular patterns (DAMPs) and tumor‐associated antigens (TAAs), thereby providing the opportunity to potentiate immunotherapies based on in situ tumor vaccines.^[^ ^4^, ^5^, ^6^, ^7^ ^]^ However, the CDT alone cannot elicit robust antitumor immune responses like tumor vaccines, and it still encounters various challenges. For instance, the targeted delivery of nano‐catalysts to tumors is often suboptimal, leading to a significant accumulation of catalytic agents in nontumor organs^[^ ^8^, ^9^ ^]^ Additionally, the effectiveness of most nano‐catalysts within the tumor microenvironment is limited in duration.^[^ ^10^ ^]^ Coupled with the insufficient concentration of endogenous H_2_O_2_ within tumors, it becomes challenging to sustain a prolonged and substantial generation of ·OH.^[^ ^11^, ^12^ ^]^ Therefore, enhancing the efficiency of ROS production within tumors and augmenting the immune responses triggered by ROS are key for enhancing the antitumor effectiveness of CDT.

However, current research on CDT primarily focuses on enhancing the catalytic efficiency of Fenton reactions through the structural design of nano‐catalysts. For instance, Professor Wenbo Bu, the pioneer of CDT, recently proposed a novel heterojunction nanoparticle to drive electron migration using built‐in electric fields for long‐term chemical dynamic therapy of tumors.^[^ ^13^ ^]^ There is also research aimed at improving the efficacy of CDT by loading glucose oxidase (GOx) onto nano‐catalysts to increase intratumoral H_2_O_2_ levels.^[^ ^14^, ^15^, ^16^ ^]^ However, the intravenous administration of nanomaterials containing GOx carries the risk of systemic toxicity.^[^ ^17^ ^]^ Furthermore, challenges remain in achieving effective tumor targeting with nano‐catalysts for CDT, as well as addressing potential issues of excessive metal ion release and associated metabolic toxicity.^[^ ^18^, ^19^ ^]^ As a potential solution, there is growing interest in hydrogel‐mediated sustained release of catalysts for Fenton reactions within tumors.^[^ ^20^, ^21^ ^]^ However, this approach might face limitations in effectively eradicating metastatic tumors. In recent years, producing in situ vaccines in tumors has demonstrated effectiveness in activating specific anti‐tumor immune responses, thereby combating metastatic tumors.^[^ ^22^, ^23^, ^24^ ^]^ Considering that CDT can induce the release of TAAs, the coexistence of immune adjuvants alongside efficient ROS generation could lead to the production of in situ tumor vaccines. This necessitates hydrogels to subtly integrate the highly effective catalysts for CDT and immune adjuvants, delivering them into tumors.^[^ ^25^, ^26^ ^]^

**Scheme 1:**
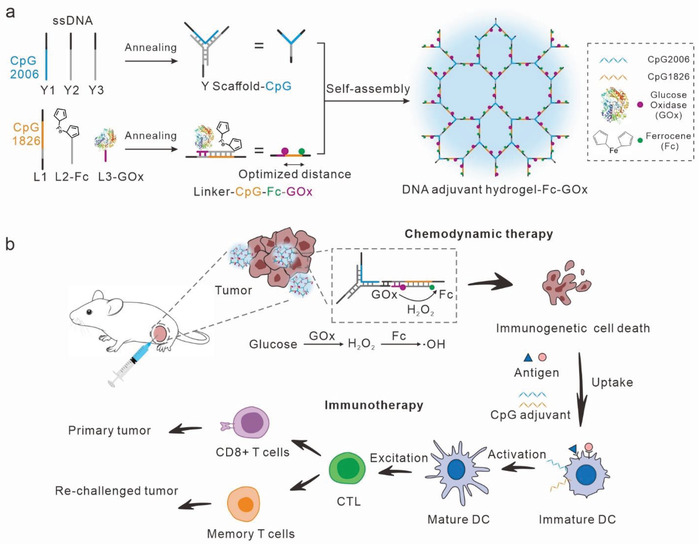
DNA adjuvant hydrogel‐scaffolded enzyme cascade reaction for tumor CDT and immunotherapy. a) Schematic illustration of self‐assembly DNA adjuvant hydrogel‐Fc‐GOx. b) After intratumor injection, highly toxic •OH can be generated under the enzyme cascade reaction of GOx and Fc for CDT. The tumor cells underwent CDT and released antigens systemic immune response induced by DNA adjuvant hydrogel‐Fc‐GOx to eliminate metastatic tumors.

DNA hydrogel is an injectable 3D network structure formed by DNA strands through physical or chemical crosslinking in an aqueous solution.^[^ ^27^, ^28^, ^29^, ^30^, ^31^ ^]^ It has superior biocompatibility and biodegradability than hydrogels based on synthetic polymers and other natural polymers (e.g., alginic acid, chitosan)^32^, ^33^ ^]^ Notably, oligodeoxynucleotides containing CpG motifs (CpG ODNs), a clinical adjuvant, are also DNA molecules and thus can be used as components of DNA hydrogels.^[^ ^34^, ^35^, ^36^ ^]^ In this work, we developed a DNA adjuvant hydrogel containing CpG ODNs to integrate GOx‐ferrocene (Fc) cascade for tumor chemodynamic‐immunotherapy. As shown in **Scheme** **1**a, two types of CpG ODNs are involved in the formation of a Y‐shaped scaffold and a linear linker within DNA hydrogels. GOx and Fc are respectively integrated into DNA adjuvant hydrogels through coupling with the DNA strands participating in the formation of the linear linker. Leveraging the programmability of DNA, we could precisely control the distance between GOx and Fc by adjusting the lengths of the two DNA strands connected to the catalysts, thereby enhancing the efficiency of the enzyme cascade reaction. We observed that, while maintaining the stability of the linear linker, setting the distance between GOx and Fc as 6.28 nm could realize the highest efficiency of glucose conversion to hydroxyl radicals, thereby effectively boosting the immunogenic cell death (Scheme 1b). Additionally, the DNA adjuvant hydrogel formulation enables sustained release of the catalyst within the body for over 4 days, facilitating the continuous generation of hydroxyl radicals for tumor cell eradication. Upon intratumoral injection, our DNA adjuvant hydrogel, incorporating enzyme cascades, proficiently suppresses the growth of localized tumors and even eliminates them. Furthermore, this chemodynamic‐immunotherapy substantially stimulated anti‐tumor immune responses, consequently restraining tumor recurrence and metastasis. This study introduces a novel platform for the synergistic combination of chemodynamic therapy and tumor immunotherapy.

## 2 Result

### 2.1 Design, Characterization, and Catalytic Performance of DNA Adjuvant Hydrogels‐Scaffolded CDT System

We assembled DNA hydrogels using Y‐shaped and cross‐linker monomers that hybridize in a 1:1.5 molar ratio, as previously reported. The Y‐shaped structure formed through the hybridization of three strands, namely Y1, Y2, and Y3. The DNA linker comprised L1, L2, and L3 strands. To construct DNA adjuvant hydrogel, we integrated CpG ODN 2006 into the Y1 strands and CpG ODN 1826 into the L1 strands. To incorporate the GOx‐Fc enzyme cascade reactions into the hydrogel, we covalently attached GOx to the 5′ end of the L3 strand (Figure S1, Supporting Information), and the 5′ end of L2 strand carried a ferrocene molecule (Figure S2, Supporting Information). Both of these strands hybridized with the L1 strand, resulting in the construction of a DNA adjuvant hydrogel‐supported CDT system (Scheme 1a).

**Figure 1:**
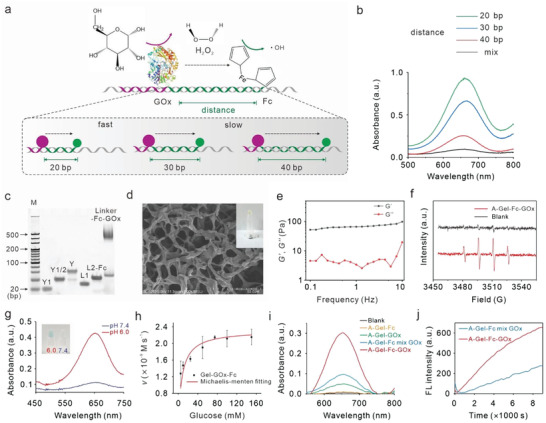
Synthesis and characterization of DNA hydrogel scaffolded catalytic performances. a) Schematic of the nanostructured complex consisting of GOx and Fc organized on a double‐stranded DNA linker. b) Distance‐dependent enzyme activity for complexes in which the distances of 20, 30, and 40 bp, as well as the mixture of two enzymes. c) PAGE characterization of Y‐shaped and cross‐linker monomers. d) SEM image of A‐Gel‐Fc‐GOx. e) Rheology analysis of A‐Gel‐Fc‐GOx. f) ESR spectra of A‐Gel‐Fc‐GOx with the addition of 25 mm glucose. g) The catalytic performances of A‐Gel‐Fc‐GOx under neutral (pH 7.4) and acidic (pH 6.0) conditions. h) Michaelis‐Menten steady‐state kinetics of DNA hydrogel supported catalytic performances. Data are presented as mean ± SD. (*n* = 3). i) UV/vis absorption spectra of TMB solution in pH 6.0 buffer with different treatments as indicated. j) Fluorescence spectra of HPF solution with GOx mixed or hybridized with DNA hydrogel‐Fc.

In our design, GOx initially converted β‐D‐glucose into β‐D‐glucono‐1,5‐lactone and H_2_O_2_, then the intermediate H_2_O_2_ was catalyzed into highly toxic ·OH by Fc under a tumorous acidic pH environment. According to previous research, the multi‐enzyme complexes show a distance‐dependent activity.^[^ ^37^, ^38^, ^39^ ^]^ To efficiently produce ·OH, we first investigated the optimal distance between GOx and Fc. By varying the length of L2, the distances between GOx and Fc were set to 20, 30, and 40 bp, respectively, to maintain consistent directionality of Fc at different distances (**Figure** **1**a; Figure S3, Supporting Information). Then, the ·OH generation catalyzed by the GOx‐Fc enzyme cascade was measured using 3,3′,5,5′‐tetramethyl‐benzidine (TMB), because ·OH can oxidize colorless TMB into chromogenic TMB cation‐free radicals with a characteristic absorbance at 650 nm. After a 2 h reaction in 25 mm glucose substrates, we discovered that the maximum yield of ·OH was obtained when GOx and Fc were separated by 20 bp, and the yields decreased sequentially at 30 and 40 bp. When the spacing between GOx and Fc was 20 bp (≈6.28 nm), the production efficiency of ·OH was elevated by up to nine times compared to a simple mixture of GOx and Fc (Figure 1b). Therefore, the L2 length was set to 20 bp in the DNA hydrogel system.

We then verified the construction of the DNA adjuvant hydrogel‐scaffolded CDT system. The assembly of Y‐shaped scaffolds and linear cross‐linkers was characterized by polyacrylamide gel electrophoresis (PAGE) (Figure 1c). Following that, scanning electron microscopy (SEM) was used to describe the structure of the DNA adjuvant hydrogels scaffolded CDT system (A‐Gel‐Fc‐GOx), revealing a cross‐linked network structure of the hydrogels (Figure 1d; Figure S4, Supporting Information). Rheology analysis of the hydrogels indicated that the shear‐storage modulus (*G*′) was higher than the shear‐loss modulus (*G*″), confirming their typical hydrogel properties (Figure 1e).

Then we evaluated the catalytic performance of these GOx‐Fc enzyme cascade reactions in DNA adjuvant hydrogels. We first determined the generation of ·OH using electron spin resonance (ESR) spectroscopy. The results showed that GOx and Fc assembled in DNA hydrogels could catalyze glucose into ·OH under an acidic environment (pH 6.0), as evidenced by the characteristic 1:2:2:1 ·OH signals in the ESR spectrum (Figure 1f). We then evaluated the catalytic performance under acidic (pH 6.0) and neutral (pH 7.4) conditions to confirm the specificity of this enzyme cascade reaction. The absorbance of TMB was almost four‐fold higher in acidic conditions than in neutral conditions, indicating that this reaction was highly specific to the acidic tumor environment (Figure 1g). The catalytic performances under acidic conditions were evaluated using the typical Michaelis–Menten steady–state kinetic. The Michaelis‐Menten constant (*K*_M_) and the max velocities (*V*_max_) of the GOx‐Fc cascade reaction were calculated to be 7.60 mm and 2.32 × 10^−9^ m s^−1^, respectively (Figure 1h), suggesting that this cascade reaction has slow but favorable steady‐state kinetics, making it suitable for slow‐release systems.

At last, we investigated whether assembling GOx into DNA hydrogels promotes ·OH production more effectively. As shown in Figure 1i, assembling GOx into DNA adjuvant hydrogel‐Fc (A‐Gel‐Fc‐GOx) significantly tripled free radical generation compared to just combining GOx with DNA adjuvant hydrogel‐Fc (A‐Gel‐Fc mix GOx). Concurrently, we used hydroxyphenyl fluorescein (HPF) to track the kinetics of both reactions. The results showed that assembling GOx into A‐Gel‐Fc boosted reaction efficiency by roughly threefold (Figure 1j). These data indicated that our DNA hydrogel‐scaffolded enzyme cascade reactions could efficiently generate ·OH in an acidic microenvironment.

### 2.2 In Vitro Cytotoxic Effect of DNA Adjuvant Hydrogel‐Optimized Enzyme Cascade Reaction

**Figure 2:**
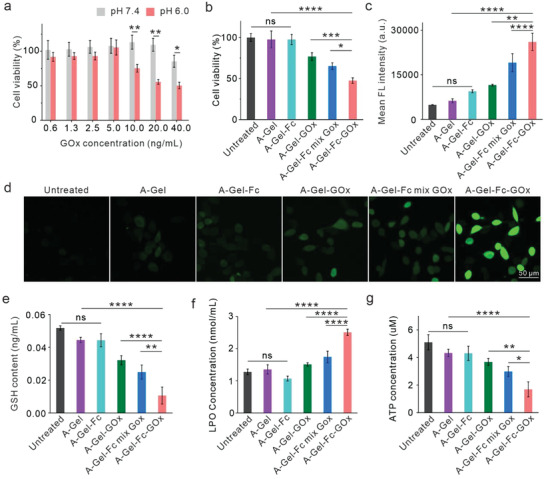
In vitro cytotoxicity and chemodynamic therapy analysis. a) Cell viability analysis of A‐Gel‐Fc‐GOx treated cells at different concentrations for 24 h. b) Cell viability analysis of A‐Gel, A‐Gel‐Fc, A‐Gel‐GOx, A‐Gel‐Fc mix GOx, and A‐Gel‐Fc‐GOx. c) Flow cytometry and d) fluorescence microscope images of B16F10 cells after incubation with different treatments for 12 h and subsequently stained with ROS fluorescence probe DCFH‐DA. Scale bar, 50 µm. e) GSH, f) LPO, and g) ATP content of B16F10 cells after different treatments. Data are presented as mean ± SD (*n* = 3). Statistical significance was calculated via one‐way ANOVA with Tukey's multiple comparisons test, ^*^ *p* < 0.05, ^**^ *p* < 0.01, ^***^ *p* < 0.001, ^****^ *p* < 0.0001.

Inspired by the excellent catalytic performance of DNA hydrogel scaffolded GOx‐Fc (Gel‐GOx‐Fc) cascade reaction in vitro, we intended to evaluate its inhibitory effect on tumor cells. First, mouse melanoma B16F10 cells were treated with A‐Gel‐GOx‐Fc at series final concentrations of GOx (40, 20, 10, 5, 2.5, 1.3, 0.6 ng mL^−1^) in both neutral (pH 7.4) and acidic (pH 6.0) cell culture media for 24 h. Cytotoxicity was evaluated using the cell‐counting kit‐8 (CCK‐8) assay. Under acidic conditions, cell viabilities decreased with increasing GOx concentration, reaching a minimum of 55.7% at 20 ng mL^−1^ GOx, but cell viabilities were substantially higher at equivalent GOx concentration under neutral conditions (**Figure** **2**a). We used a final GOx concentration of 20 ng mL^−1^ in the following cell studies. Further research revealed that A‐Gel and the integration of Fc alone into A‐Gel (A‐Gel‐Fc) had little effect on cell viability. A‐Gel integrating GOx alone (A‐Gel‐GOx) resulted in just a modest decrease in cell viability (76.8%), probably due to the production of weakly oxidizing H_2_O_2_. The viability of cells treated with A‐Gel‐GOx‐Fc was significantly lower than that treated with A‐Gel‐Fc containing free GOx (A‐Gel‐Fc mix GOx) (47.5% vs 65.2%), indicating that the cascade reaction caused by the co‐assembly of GOx and Fc at a certain distance could result in greater cytotoxicity (Figure 2b).

Following that, we evaluated the oxidative stress induced by the generated ·OH in B16F10 cells. The ·OH level in cells was detected using 2′,7′dichlorofluorescin diacetate (DCFH‐DA) staining. Cells treated with A‐Gel, A‐Gel‐Fc, A‐Gel‐GOx, and A‐Gel‐Fc mix GOx barely showed any fluorescence, while A‐Gel‐GOx‐Fc displayed remarkably enhanced green fluorescence, confirming its superior ·OH generation capabilities (Figure 2c,d). The intracellular glutathione (GSH) and lipid hydroperoxide (LPO) levels were also measured after different treatments. As shown in Figure 2e,f, A‐Gel‐GOx‐Fc could more significantly down‐regulated the GSH level and promote the LPO generation. In addition, as shown in Figure 2g, the ATP level in tumor cells treated by A‐Gel‐GOx‐Fc was the lowest, partially due to the blocking of glycolysis by the depletion of glucose. All these results indicated that A‐Gel‐GOx‐Fc could efficiently induce oxidative stress aggravation in tumor cells.

### 2.3 ICD Induced by DNA Adjuvant Hydrogel‐Optimized Enzyme Cascade Reaction

**Figure 3:**
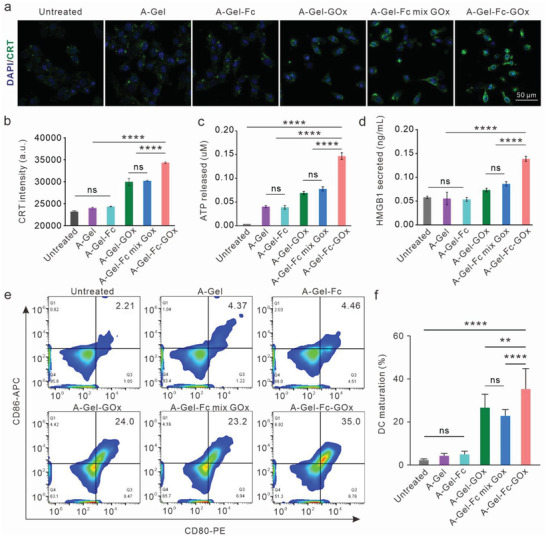
In vitro immunogenic cell death triggered by DNA hydrogel‐supported CDT. a) Fluorescence imaging of CRT expression on the B16F10 cell surface after being treated with different DNA hydrogels. Cell nuclei were stained with DAPI (blue); CRT was stained with Alexa‐488‐conjugated anti‐CRT antibody (green). Scale bar: 50 µm. b) Flow cytometry quantification of CRT on the surface of B16F10 cells. c) ATP secretion detected by an enhanced ATP assay kit. d) HMGB1 secretion detected by an enzyme‐linked immunosorbent assay (ELISA) kit (*n* = 3). e) Flow cytometry analysis of DC maturation (gated on CD11c^+^ cells) after being incubated with cell medium from different DNA hydrogels treated B16F10 cells. f) Statistical analysis of e). Data are presented as mean ± SD. (*n* = 3). Statistical significance was calculated via one‐way ANOVA with Tukey's multiple comparisons test, ^**^ *p* < 0.01, ^***^ *p* < 0.001, ^****^ *p* < 0.0001.

Chemodynamic therapy has been demonstrated to destroy cancer cells directly while also inducing ICD, leading to the release of TAAs and DAMPs that activate dendritic cells (DCs) and boost antitumor immunity. Subsequently, we investigated the efficacy of DNA hydrogel‐scaffolded CDT system in inducing ICD with B16F10 cells, characterized by calreticulin (CRT) exposure, high mobility group box 1 (HMGB1) release, and ATP secretion.^[^ ^40^ ^]^ As shown in **Figure** **3**a, confocal imaging revealed CRT expression on the surface of the B16F10 cell membrane. Notably, A‐Gel‐GOx‐Fc treatment resulted in more remarkable CRT exposure on the cell membrane compared to A‐Gel‐Fc mix GOx and A‐Gel‐GOx under identical conditions (Figure 3b). Moreover, A‐Gel‐GOx‐Fc could more significantly induce HMGB1 release and ATP secretion (Figure 3c,d).

To assess the potential of ICD in promoting DC maturation, we exposed bone marrow‐derived dendritic cells (BMDCs) to the cell medium from A‐Gel, A‐Gel‐Fc, A‐Gel‐GOx, A‐Gel‐Fc mix GOx, and A‐Gel‐GOx‐Fc treated B16F10 cells, respectively. After treatment for 12 h, the DCs were collected and analyzed by flow cytometry to measure the expression of CD86 and CD80. As shown in Figure 3e,f, A‐Gel‐GOx‐Fc significantly enhanced the maturation of DCs (CD86^+^/CD80^+^, gated on CD11c^+^ cells) to 35.3%, much higher than that in both A‐Gel‐GOx and A‐Gel‐Fc mix GOx groups (Figure S5, Supporting Information). All the above results demonstrated that A‐Gel‐GOx‐Fc could efficiently induce ICD, thus promoting DC maturation.

### 2.4 Efficacy of in Situ Chemodynamic‐Immunotherapy on Mouse Colon Cancer Model

**Figure 4:**
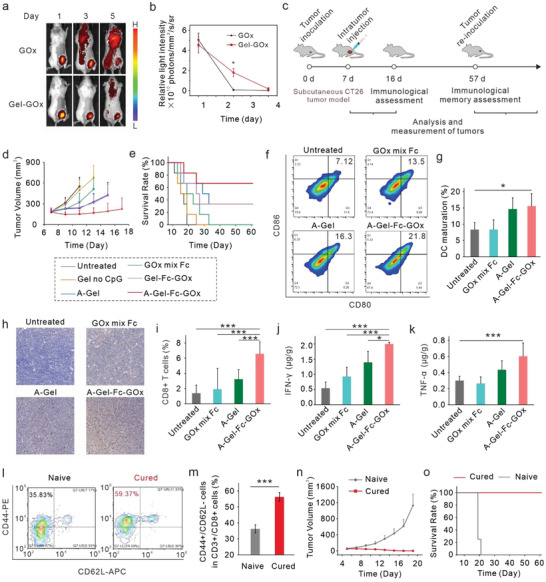
In vivo, antitumor efficacy of CDT combined with immunotherapy based on DNA hydrogel for treatment colon tumors. a) In vivo fluorescence imaging of the distribution of free GOx and DNA hydrogel‐loaded GOx. b) Statistical results corresponding to a). The error bars represent mean ± SD (*n* = 4). c) Schematic illustration of CDT combined with immunotherapy based on DNA hydrogel for the treatment of CT26 murine colon tumors and immunological memory assessment. d) Tumor growth curves on CT26‐tumor‐bearing mice with different treatments as indicated. The error bars represent mean ± SD (*n* = 6). e) Survival rates of CT26‐tumor‐bearing mice with different treatments as indicated. f) Flow cytometry results showing the percentages of DC maturation levels following different treatments. g) Statistical results corresponding to d). The error bars represent mean ± SD (*n* = 4). h) Immunohistochemical staining of CD8+ T cells on tumor sections. i) Statistical results corresponding to h). The error bars represent mean ± SD (*n* = 4). j) IFN‐γ levels and k) TNF‐α levels in serum measured 16 days later after different treatments. The error bars represent mean ± SD (*n* = 4). l) Represent flow cytometry results showing the percentages of Tem among CD8+ T cells of naive mice (left) and cured mice (right). m) Statistical results corresponding to l). The error bars represent mean ± SD (*n* = 4). n) Tumor growth curves of the re‐challenged tumors on naive mice and cured mice. The error bars represent mean ± SD (*n* =  4). o) Survival rate of the naive mice and cured mice. Statistical significance was calculated via one‐way ANOVA with Tukey's multiple comparisons test. ^*^ *p* < 0.05, ^**^ *p* < 0.01, ^***^ *p* < 0.001.

DNA hydrogels have shown great potential as carriers for sustained therapeutic delivery.^[^ ^41^, ^42^ ^]^ The hydrogel exhibited outstanding injectability, smoothly passing through a 1 mL syringe, and maintained its cross‐linked network structure post‐injection (Figure S6, Supporting Information). We first evaluated the local retention time of DNA hydrogel‐loaded GOx in tumor. For this purpose, GOx conjugated with the L3 DNA strand was labeled with Cy5. Then, fluorescently labeled GOx loaded by DNA hydrogel were injected intratumorally into the CT26 mouse model, with free GOx as control, respectively. According to the in vivo fluorescence imaging and statistical results, GOx loaded in DNA hydrogels showed significantly longer retention time than free GOx (**Figure** **4**a,b), suggesting that the enzymes assembled in DNA hydrogels could produce ·OH continuously in the tumor.

We then utilized DNA adjuvant hydrogel‐scaffolded enzyme cascade reactions to combine CDT with immunotherapy in the treatment of mouse tumors. Treatment began 7 days following inoculation with CT26 colon tumors, as illustrated in Figure 4c. BALB/c mice bearing colon tumors were divided into 6 groups (*n* = 6) randomly: Group 1, Untreated; Group 2, Gel no CpG; Group 3, A‐Gel; Group 4, GOx mix Fc; Group 5, Gel‐GOx‐Fc; Group 6, A‐Gel‐GOx‐Fc. In groups 2 and 5, the CpG ODN sequences in the DNA hydrogels are nonphosphorothioate modified, thus having dramatically decreased adjuvant activity. In group four, free GOx and Fc were simply mixed for i.t. injection. After injecting the above‐described formulations into tumors, the tumor volume and body weight of the mice were recorded every 2 days. According to the tumor growth curve (Figure 4b) and survival data of the mice (Figure 4c), we found that the mixture of GOx and Fc (group 4) showed limited effect in suppressing tumor growth and prolonging animal survival. This is attributed to the rapid clearance of free GOx and Fc from the tumor. In contrast, the GOx and Fc assembled in the DNA hydrogel in group 5 were sustained and released in TME, maintaining a relatively high concentration of ROS for a long time, thereby enhancing the therapeutic effect. Of note, the CpG ODN sequences in groups 2 and 5 were nonphosphorylated, resulting in less efficacy compared to groups 3 and 6, possibly due to the inability to stimulate the immune system. Remarkably, A‐Gel‐CpG‐GOx‐Fc (group 6) successfully suppressed tumor growth, with four out of six tumors eliminated. Because of the good biocompatibility of this therapy, mouse body weights were not significantly affected by the treatment (Figure S7, Supporting Information).

Next, we explored the mechanism of the above combination therapy by studying the immune response after different treatments. DCs are able to present tumor antigens to CD8^+^ T cells, inducing the activation and proliferation of antigen‐specific CD8^+^ T cells for the specific killing of tumor cells. Therefore, on the 9th day after treatments, mice from each group were sacrificed to evaluate the maturation of DCs in the inguinal lymph nodes and the infiltration rate of CD8^+^ T cells in the tumors. Although GOx mix Fc and A‐Gel could promote the DC maturation, A‐Gel‐Fc‐GOx significantly increased the maturation of DCs (Figure 4f,g) and the infiltration of CD8^+^ T cells in the tumors (Figure 4h,i). In addition, we examined the levels of cytokines related to cellular immunity in tumors, including necrosis factor alpha (TNF‐α) and interferon‐gamma (IFN‐γ). Results showed that A‐Gel‐Fc‐GOx could significantly increase the secretion levels of these cytokines in the tumors (Figure 4j,k), further demonstrating the enhanced cytotoxic functions of CD8^+^ T cells. Our results demonstrated that the DNA adjuvant hydrogel‐scaffolded enzyme cascade reaction could dramatically enhance the efficacy of CDT by promoting antitumor immune responses.

Immunological memory provides rapid protection against pathogen re‐infection and tumor recurrence. To verify immunological memory induced by DNA adjuvant hydrogel‐scaffolded enzyme cascade reaction, we collected peripheral blood from the cured mice 50 days after the initiation of our treatment to identify effector memory CD8^+^ T cells (Tem, CD3^+^CD8^+^CD62L^−^CD44^+^) (Figure 4l,m; Figure S8, Supporting Information). The percentage of CD8^+^ Tem cells in the peripheral blood of the cured mice was significantly higher than that of untreated mice (59.37% vs 35.83%). We then re‐inoculated CT26 colon tumors into the cured mice. We observed complete inhibition of tumor growth as the result of strong immunological memory in A‐Gel‐Fc‐GOx cured mice (Figure 4n), and survival was significantly prolonged compared with naive mice (Figure 4o). The above findings are strong evidence that chemotherapy assisted with DNA adjuvant hydrogel‐scaffolded enzyme cascade reaction would be capable of inducing excellent immunological memory effects to effectively impede tumor recurrence.

To evaluate the potential damage induced by DNA adjuvant hydrogel‐Fc‐GOx, we performed histological analysis on various tissues, revealing no apparent organ damage in mice (Figure S9, Supporting Information). Additionally, we examined the local skin and muscle tissues at the injection site using tissue section H&E staining, demonstrating that hydrogel treatment did not cause damage to the local skin and muscle (Figure S10, Supporting Information).

### 2.5 Efficacy of in Situ Chemodynamic‐Immunotherapy on Mouse Melanoma and Breast Cancer Models

**Figure 5:**
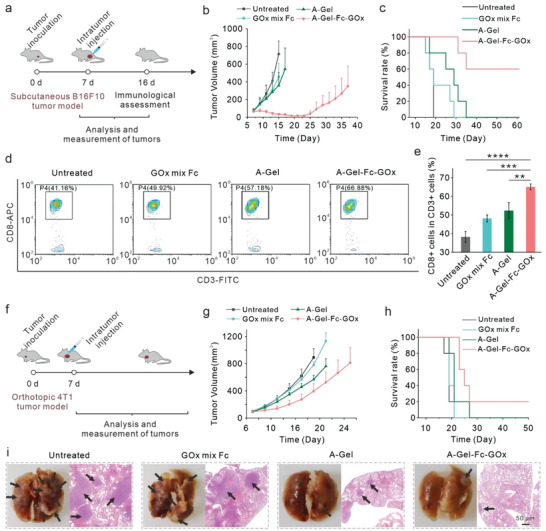
In vivo antitumor efficacy of CDT combined with immunotherapy based on DNA hydrogel for the treatment of subcutaneous B16F10 melanoma tumors and orthotopic 4T1 breast tumors. a) Schematic illustration of CDT combined with immunotherapy based on DNA hydrogel for the treatment of B16F10 murine melanoma tumors and immunological assessment. b) Tumor growth curves on B16F10‐tumor‐bearing mice with different treatments as indicated. The error bars represent mean ± SD (*n* = 6). c) Survival rates of B16F10‐tumor‐bearing mice with different treatments as indicated. d) Flow cytometry results showing the percentages CD8+ T cell maturation levels following different treatments. e) Statistical results corresponding to d). The error bars represent mean ± SD (*n* = 4). Statistical significance was calculated via one‐way ANOVA with Tukey's multiple comparisons test. ^**^ *p* < 0.01, ^***^ *p* < 0.001, ^****^ *p* < 0.0001. f) Schematic illustration of CDT combined with immunotherapy based on DNA hydrogel for orthotopic 4T1 breast cancer treatment. g) Tumor growth curves on 4T1‐tumor‐bearing mice with different treatments as indicated. The error bars represent the mean ± standard error of the mean (*n* = 6). h) Survival rates of 4T1‐tumor‐bearing mice with different treatments as indicated. i) Representative photographs and H&E staining of the lung tissues of the 4T1‐tumor‐bearing BALB/c mice collected on the 27th‐day post. The black arrows indicated the presence of metastatic lesions of the 4T1 breast tumor in the lung.

To test the universality of this combination of CDT and immunotherapy in enhancing the therapeutic outcome of different kinds of tumors, we first tested our strategy on subcutaneous B16F10 malignant melanoma. Treatment started on day 7, followed by recording the tumor volume and body weight of the mice every two days (**Figure** **5**a). C57 mice bearing B16F10 tumors were divided into 4 groups: Group 1, untreated; Group 2, GOx mix Fc; Group 3, A‐Gel; Group 4, A‐Gel‐Fc‐GOx. As shown by the tumor growth curves and survival data (Figure 5b,c), i.t. injection of A‐Gel‐Fc‐GOx could also significantly enhance the tumor suppression effect and result in much‐prolonged animal survival time. Also, no appreciable body weight drop was observed (Figure S11, Supporting Information). In addition, we found a higher CD8^+^ T cells ratio (66.68%) in T cells of peripheral blood was achieved in the A‐Gel‐Fc‐GOx treatment group compared with other groups (Figure 5d,e; Figure S12, Supporting Information), suggesting the enhanced cellular immunity in tumor‐bearing mice.

Finally, we validated the anti‐tumor metastatic ability of the combination treatment on the 4T1 orthotopic tumor model. Treatment started on day 7 after tumor inoculation, and the tumor sizes and weight of mice were recorded simultaneously (Figure 5f). Tumor‐bearing mice were divided into 4 groups: Group 1, untreated; Group 2, GOx mix Fc; Group 3, A‐Gel; Group 4, A‐Gel‐GOx‐Fc. As shown by the tumor growth curve (Figure 5g), A‐Gel‐GOx‐Fc treatment (group 4) showed more efficient tumor growth inhibition than that treated by free GOx and Fc (group 2) or A‐Gel (group 3). Remarkably, we observed a largely prolonged survival (Figure 5h) and no significant body weight change (Figure S13, Supporting Information) in the A‐Gel‐GOx‐Fc treatment group. As the 4T1 tumor can spontaneously metastasize from the primary tumor to the lymph nodes, brain, lung, liver, and bone, we further obtained the lungs of the mouse of which the primary tumor volume exceeded 1000 mm^3^ on the 27th day for counting metastatic nodules. The representative lung photographs showed that while there were dense metastatic nodules (indicated by the black arrow) in the lungs of untreated mice, A‐Gel‐GOx‐Fc treatment could significantly inhibit tumor metastasis (Figure 5i). The pathological changes of the representative lung tissues were further observed in hematoxylin‐eosin (H&E) staining, which also verified that A‐Gel‐GOx‐Fc treatment could dramatically suppress lung metastases of 4T1 tumors (Figure S14, Supporting Information).

## 3 Conclusion

In summary, we have developed a DNA adjuvant hydrogel‐optimized enzyme cascade for efficient chemodynamic therapy combined with immunotherapy, achieving the effective treatment of various solid tumor models on mice. Leveraging the high programmability of DNA, and precise control of the spacing between glucose oxidase and ferrocene in this enzyme cascade reaction has significantly improved the efficiency of generating ROS, leading to more efficient tumor cell death. After intratumoral administration, the combination of immunoadjuvants continuously released from DNA hydrogels and tumor‐associated antigens released from immunogenic cell death of tumor cells can significantly inhibit tumor growth, recurrence, and metastasis. Therefore, by promoting the synergistic of CDT and immunotherapy, this DNA adjuvant hydrogel‐optimized enzyme cascade holds great potential in the treatment of solid tumors.

## Conflict of Interest

The authors declare no conflict of interest.

## Supporting information

Supporting Information

## Data Availability

The data that support the findings of this study are available in the supplementary material of this article.
