# Supplementary material for: DNA Adjuvant Hydrogel‐Optimized Enzymatic Cascade Reaction for Tumor Chemodynamic‐Immunotherapy
Source: Adv Sci (Weinh). 2024 Jan 15;11(10):2308229. doi: 10.1002/advs.202308229 (PMC10933675; doi:10.1002/advs.202308229)
Supplement: Supplementary file 1 — Supporting Information [file ADVS-11-2308229-s001.pdf]

ADVANCED SCIENCE
Open Access

Supporting Information

for *Adv. Sci.*, DOI 10.1002/advs.202308229

DNA Adjuvant Hydrogel-Optimized Enzymatic Cascade Reaction for Tumor Chemodynamic-Immunotherapy

Yan Zhao, Jiangnan Du, Zihui Xu, Lihua Wang, Lan Ma and Lele Sun**

Experimental Procedures

Materials.

DNA strands were synthesized and purified by Sangon Biotech (Shanghai) Co., Ltd. Glucose oxidase and Sulfo-SMCC were purchased from Sigma-Aldrich. Tris(2-carboxyethyl)phosphine (TCEP) was purchased from Aladdin. Reduced glutathione (GSH) assay kit, mouse high mobility group protein 1 (HMGB-1) ELISA kit and lipid peroxide (LPO) content assay kit were obtained from Solarbio. 2’, 7’-Dichlorodihydrofluorescein diacetate (DCFH-DA), Cell counting kit-8 (CCK8) and ATP assay kit were obtained from Beyotime Biotechnology. Antibodies used for flow cytometry analysis were purchased from Biolegend and Proteintech.

The murine colon carcinoma CT26, melanoma B16F10 and breast carcinoma 4T1 cell lines were purchased from Guangzhou Xinyuan technology Co., Ltd. All cells were cultured in DMEM medium supplemented with 10% FBS and 1% penicillin/streptomycin in a humidified 37 °C and 5% CO_2 incubator.

Female C57BL/6 mice (6-8 weeks old) were purchased from Soochow University Laboratory Animal Center, and used under the animal experiment protocols approved by them (approval number: SYXK(Su) 2017-0043).

Fabrication and characterization of DNA hydrogel.

To construct glucose oxidase (GOx) encapsulated DNA hydrogel, the thiol-ssDNA (L3-SH) was conjugated with GOx. Briefly, L3-SH was mixed with Sulfo-SMCC at a molar ratio of 1:5 and shaken at room temperature for 0.5 h in PBS. Excess SMCC was removed by ultrafiltration. Next, L3-SH and GOx were mixed in PBS at a molar ratio of 1:5, then shaken at room temperature for 2 h. The successful coupling of DNA and GOx (L3-GOx) was identified by polyacrylamide gel electrophoresis (PAGE).

The DNA hydrogel was synthesized as previous studies. We took Gel-Fc-GOx as an example. Firstly, Y-scaffold (Y1/Y2/Y3) and Linker (L1/L2-Fc20) strands were mixed in buffer (100 mM Tris-HCl, pH 6.0, 250 mM NaCl) with final concentrations of 1 mM and 1.5 mM, separately. The mixture solutions were heated to 95 °C for 5 min and then slowly cooled to 4 °C within 2 h. Then, Linker was hybridized with L3-GOx at a ratio of 1:1 for 10 min at 37 °C. Finally, Y-Scaffold and Linker were mixed with a molar ratio of 2:3 to get the A-Gel-Fc-GOx. The DNA strands were listed in Table S1, and the corresponding sequences can be changed to synthesize desired DNA hydrogels (Table S2). The prepared monomers were characterized by PAGE.

Scanning electron microscopy (SEM) characterization.

A-Gel-Fc-GOx after thoroughly freeze drying under vacuum was put onto clean Silicon plates, and their morphology were examined by SEM (HITACHI, SU8010). EDS mapping was conducted for elemental distribution analysis.

Rheological analysis.

Rheological tests were carried out on an ARES-RFS rheometer with 8 mm parallel-plate geometry. The viscosity curves were recorded under rotational runs at 25°C. Time-scan test was carried out at a fixed strain of 1% and frequency of 1 Hz at 25 °C.

Catalytic performance.

12.5 μL A-Gel-Fc-GOx was added into a series of glucose concentration (40, 80, 120, 160 mM at pH 6.0), and mixed with TMB solution (2 mg/mL). The chromogenic reaction (λ = 650 nm) was determined by the UV-vis spectra at indicated time intervals. Michaelis-Menten kinetic curve could be obtained by plotting velocity against glucose concentration. The Michaelis-Menten constant (K_M) and maximal velocity V_{max} were calculated via the Lineweaver-Burk plotting.

12.5 μL of different components of DNA hydrogels (A-Gel, A-Gel-Fc, A-Gel-GOx, A-Gel-Fc mix GO, A-Gel-Fc-GOx) were mixed with glucose solution (25 mM, pH 6.0) and reacted for 20 min. The production of ·OH was evaluated by chromogenic reaction of TMB.

In vitro cytotoxicity.

B16F10 cells were seeded at a density of 1×10^4 cells per well in a 96-well plate and cultured overnight. After that the medium was changed with fresh DMEM with pH 6.0, the followed by the addition of 6.25 μL different DNA hydrogels (A-Gel, A-Gel-Fc, A-Gel-GOx, A-Gel-Fc mix GOx, A-Gel-Fc-GOx) to make a total volume of 100 μL. Each group was conducted in triplicate. After 12 h incubation, the medium was removed, and fresh medium containing 10% CCK8 was added to each well. After incubation for another 30 min, the 96-well plate was measured by a microplate reader at the absorbance of 450 nm.

A-Gel-Fc-GOx with a series concentration (depending on GOx) was dispersed in DMEM medium with pH adjusted to 6.0 or 7.4. The cell cytotoxicity was evaluated by CCK8 assay as above.

ATP assay.

B16F10 cells were cultured in a 6-well plate. In Fig. 2g, the intracellular ATP content was assessed by collecting supernatants after various treatments, followed by processing with an ATP assay kit. In Fig. 3c, the secreted ATP content was evaluated by collecting cell medium. The ATP levels were quantified using enhanced chemiluminescence measured with a microplate reader.

Intracellular ROS detection and imaging.

B16F10 cells were inoculated at 5×10^4 per well in 24-well plates and cultured overnight. Then the medium was changed to fresh DMEM with pH 6.0, following by addition of 12.5 μL different DNA hydrogels (A-Gel, A-Gel-Fc, A-Gel-GOx, A-Gel-Fc mix GO, A-Gel-Fc-GOx) to produce ROS, and PBS as control. After incubation for 12 h, the medium was removed and DCFH-DA diluted in serum-free DMEM was added to each well. Flow cytometry was used to quantify the intracellular ROS.

Using confocal microscope dishes to incubate cells and treated in the same way, CLSM (Nikon Eclipse

Ti) was used to detect the production of intracellular ROS.

In vitro DC maturation.

Bone marrow stem cells were extracted from 6-week-old female C57BL/6 mice and induced with GM-CSF to differentiate to bone marrow derived dendritic cells (BMDCs) ($CD11c^+$). DNA hydrogels treated B16F10 cell culture medium was added BMDCs separately. After 12 h incubation, the frequency of mature DCs ($CD11c^+CD80^+CD86^+$) was examined by flow cytometry (Beckman, CytoFLEX).

In vivo anti-tumor efficacy

CT26 cells (1×10^6/each) were inoculated subcutaneously in 5-week-old C57BL/6 mice to construct a colon cancer model. The mice were divided into 6 groups equally, one group was the control group using saline, and the remaining 5 groups were treated with Gel no CpG, A-Gel, GOx mix Fc, Gel-Fc-GOx, and A-Gel-Fc-GOx. At day 7, 100 μL of each sample was injected to the tumor in situ. The tumor volumes and mice body weights were monitored every other day. The survival time of the remaining mice were monitored until day 60 post the first injection. Tumor volume was calculated as the following formula:

$$\text{Tumor volume} = 0.5\times L\times W^2$$

(L: the longest dimension, W: the shortest dimension).

The mice subcutaneous melanoma and orthotopic breast cancer models were conducted as above.

In vivo immune responses

On day 16 after treatment of CT26 tumor-bearing mice with different fractions, some mice were taken from each group. The lymph nodes were harvested and filtrated through a 200-mesh filter to get single cells. The maturated DC cells ($CD11c^+CD80^+CD86^+$) were measured by flow cytometry.

To verify the intratumor infiltration of T lymphocytes, on day 16 after treatment of CT26 tumor-bearing mice with different fractions, some mice were taken from each group. The tumor tissues were collected and making into frozen slices, using IHC easy CD8 Ready-To-Use IHC (Proteintech) for immunohistochemical analysis.

To analyze the mouse cytokine and interferon responses, mice were euthanized and serum was collected after 16 days. Tumor necrosis factor α (TNF-α) and Interferon γ (IFN-γ) were measured using ELISA kits (Solarbio) according to the manufacturer's instructions.

Anti-tumor metastatic ability assay

Mouse 4T1 breast cancer lung metastasis models were constructed. The tumor-bearing mice were equally divided into 4 groups, one of which was the control, and the remaining 3 groups were treated with using straight GOx mix Fc, A-Gel, and A-Gel-Fc-GOx. At day 7, 100 μL of each was injected in situ in the tumor. The tumor volumes and mice body weights were monitored every other day. The survival time of the remaining mice for each group were monitored until day 24 post the first injection. After 24 days some mice

were taken from each group. The lungs were collected and making into frozen evaluated with Hematoxylin/eosin (H&E).

Tumor rechallenge studies

After complete cure of the mice with A-Gel-Fc-GOx, the mice were allowed to recover for 57 days and then inoculated again with CT26 cells (3×10^6/each) on the other side of the mice, as well as untreated mice. The tumor volume size of the mice was measured every other day, while the survival of each group of mice was observed until day 60 after reinoculation to test the presence of immune memory. The spleen was harvested and filtrated through a 200-mesh filter to get single cells. The effector memory T cells ($CD44^+CD62L^-$ in $CD3^+CD8^+$ cells) were measured by flow cytometry.

Statistical analysis.

All statistical analyses were conducted on Origin software. Data from the experiments were performed for three times and the results are presented as the mean ± standard deviation (SD). One-way ANOVA with Tukey's multiple comparisons test was performed for statistical analysis of the difference between the two groups. P value < 0.05 was considered statistically significant between the data sets, where all significant values were indicated as follows: *p< 0.05, **p< 0.01, ***p< 0.001.

Supporting Tables

Table S1. Strands for synthesizing DNA hydrogel

Name	Sequence (5'-3')
Y1	CGATTGACTCGTCGTTTTGTCGTTTTGTCGTT
Y2	CGATTGACAACGACAAAACGCACGCTGTCCTA
Y3	CGATTGACTAGGACAGCGTGACAAAACGACGA
Y1-S	CGATTGACT*C*G*T*C*G*T*T*T*T*G*T*C*G*T*T*T*T*G*T*C*G*T*T
L1	GTCAATCGGTGCTTGGTAACACATCCATGACGTTCCTGACGTTGCCCGGCTCTTGTAAGTCGG GTCAATCG
L2	AACGTCAGGAACGTCATGGA
L1-S	GTCAATCGGTGCTTGGTAACACAT*C*C*A*T*G*A*C*G*T*T*C*C*T*G*A*C*G*T*TGCCCGG CTCTTGTAAGTCGGGTCAATCG
L2-Fc20	Ferrocene-AACGTCAGGAACGTCATGGA
L2-Fc30	Ferrocene-AGAGCCGGGCAACGTCAGGAACGTCATGGA
L2-Fc40	Ferrocene-CCGACTTACAAGAGCCGGGCAACGTCAGGAACGTCATGGA

L3-SH	SH-TGTGTTACCAAGCA

Table S2. DNA strands used in hydrogel.

Gel Name	DNA strands
Gel	Y1, Y2, Y3, L1, L2, L3
Gel-GOx	Y1, Y2, Y3, L1, L2, L3-GOx
Gel-Fc	Y1, Y2, Y3, L1, L2-Fc20
Gel-Fc mix GOx	Y1, Y2, Y3, L1, L2-Fc20
Gel-Fc-GOx	Y1, Y2, Y3, L1, L2-Fc20, L3-GOx
Gel-CpG	Y1-S, Y2, Y3, L1-S, L2, L3
Gel-CpG-Fc-GOx	Y1-S, Y2, Y3, L1-S, L2-Fc20, L3-GOx

Supporting Figures

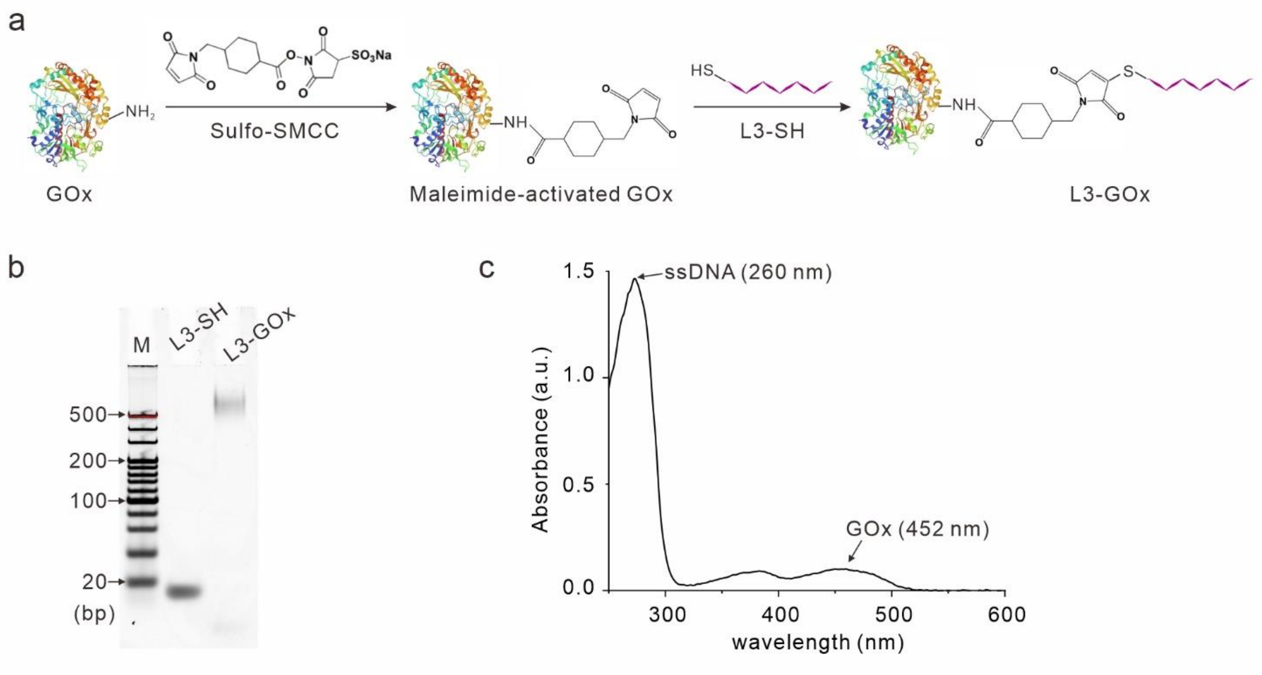

Supporting Figure 1. The conjugation of GOx and L3 ssDNA. a) The coupling process of sulfhydryl-L3 and GOx. b) PAGE analysis of L2-GOx. M: 20bp DNA ladder. c) UV-vis spectrum of L2-GOx. Based on calculations, an average of 4.5 single-stranded DNA molecules are connected to each GOx.

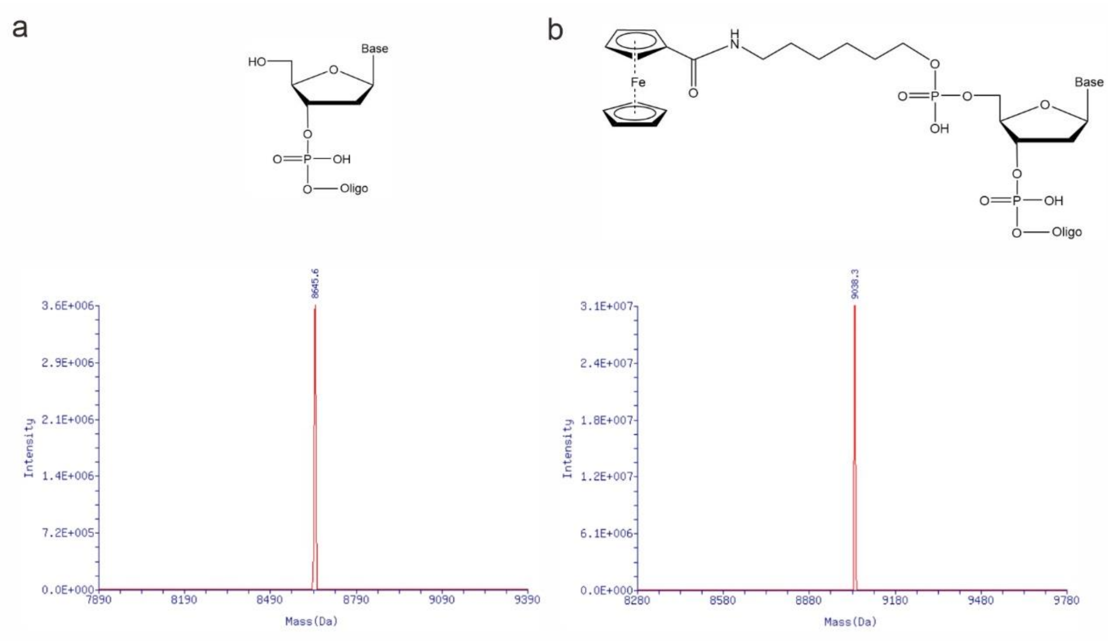

Supporting Figure 2. Structural formulas and mass spectra (MS) of DNA (a) and Fc-DNA (b). The MS results proved a molecule weight of 8645.6 and 9038.3 for DNA and Fc-DNA, with an increase of 392.7, slightly larger than theoretical value of Fc (390.7), indicated the success of Fc modification.

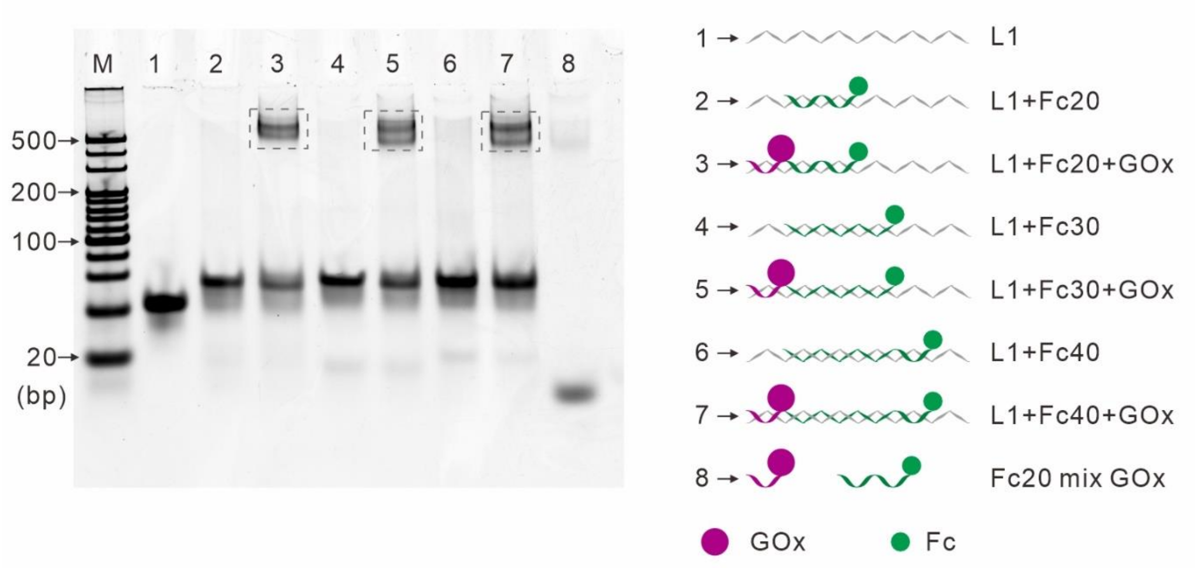

Supporting Figure 3. PAGE analysis of the assembly of GOx and Fc with different distances.

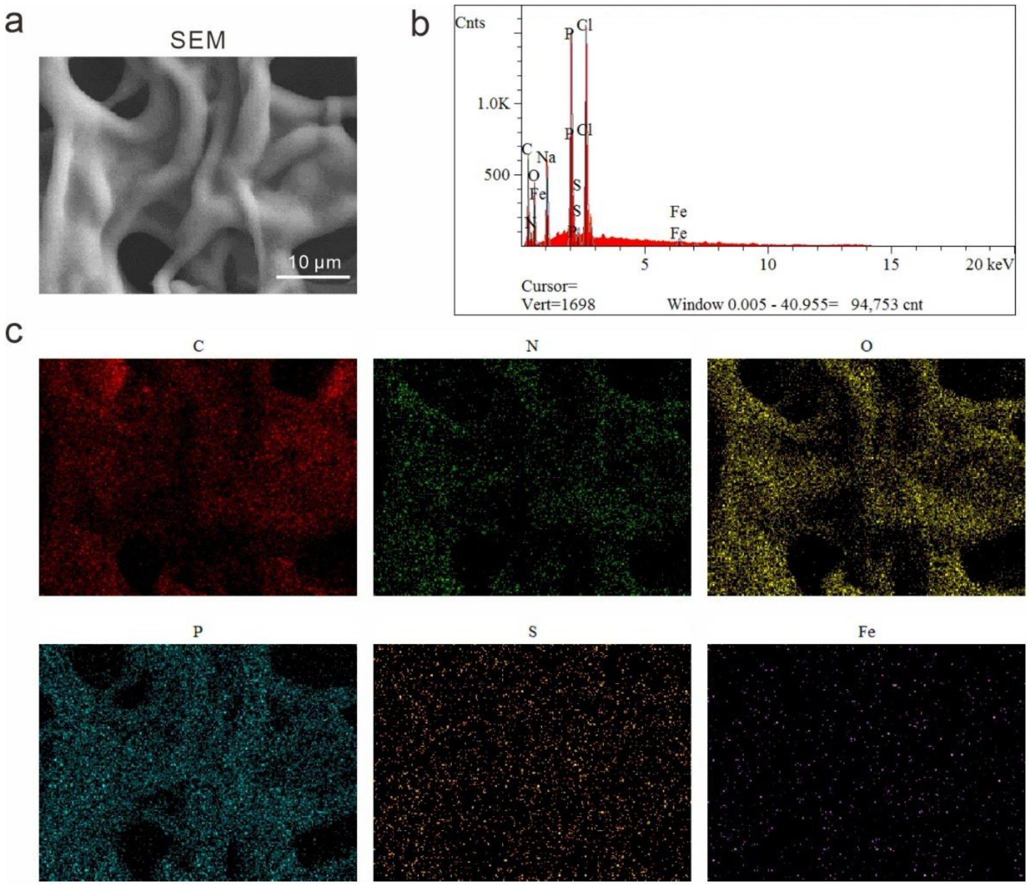

Supporting Figure 4. SEM image (a), EDS (b), and corresponding area-elemental mappings (c) of the DNA adjuvant hydrogel-Fc-GOx.

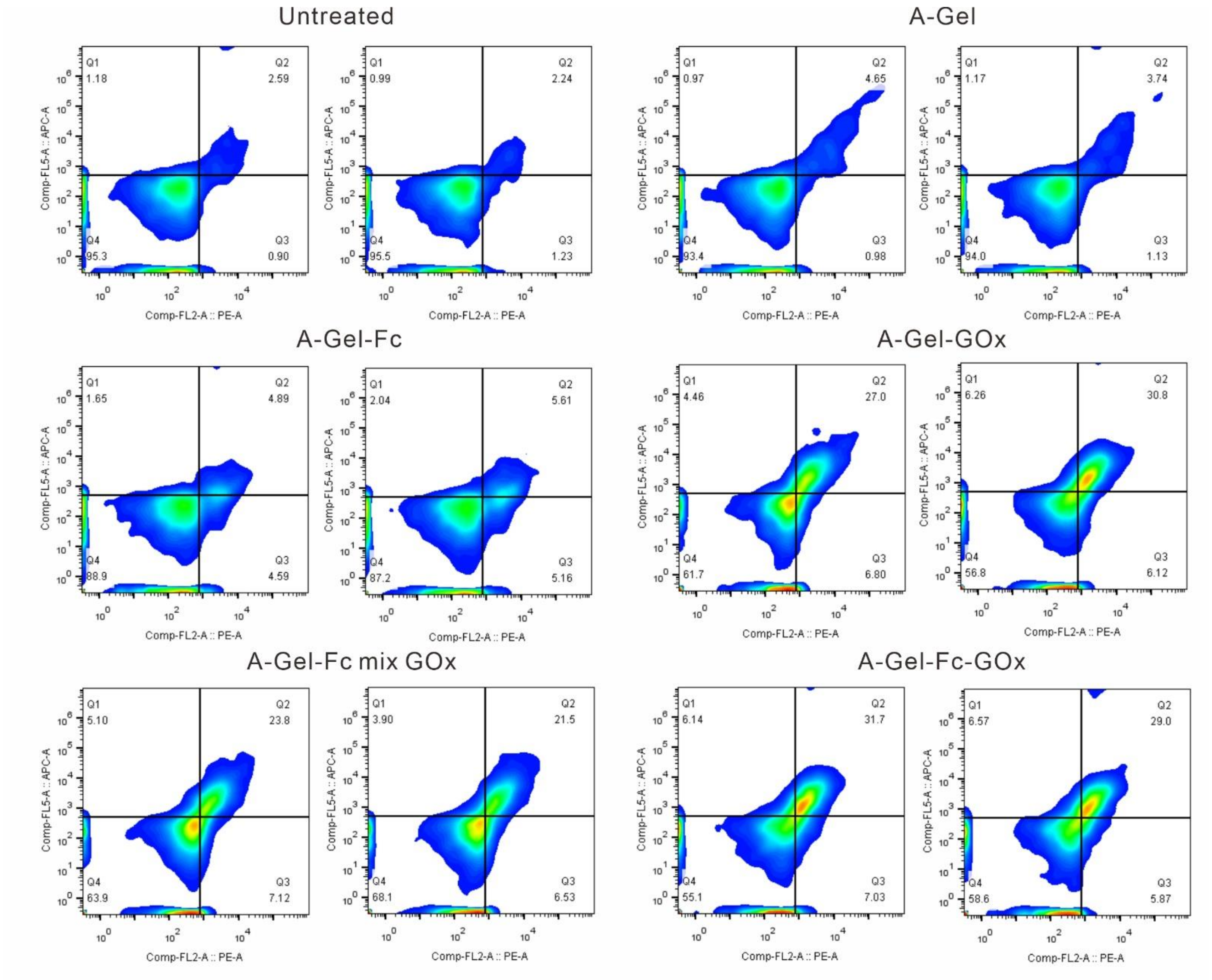

Supporting Figure 5. Flow cytometry analysis of DC maturation (gated on CD11c+ cells) after being incubated with cell medium from different DNA hydrogels treated B16F10 cells.

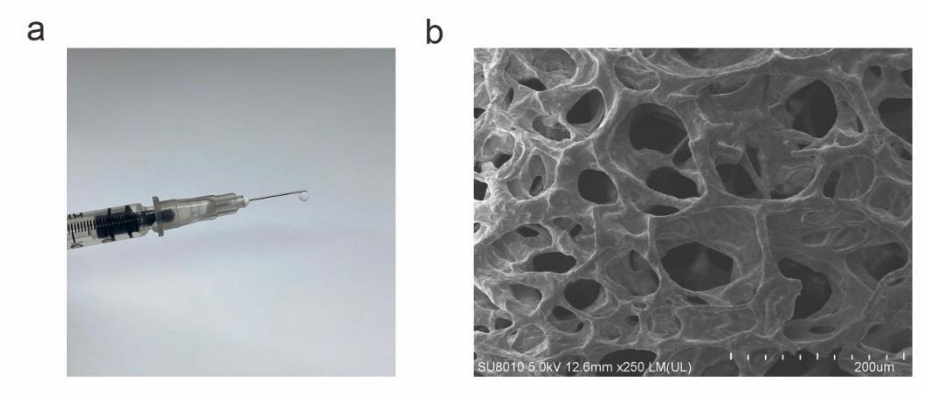

Supporting Figure 6. (a) The DNA adjuvant hydrogel-Fc-GOx was injected using a 1 mL syringe. (b) SEM image of the hydrogel post-injection.

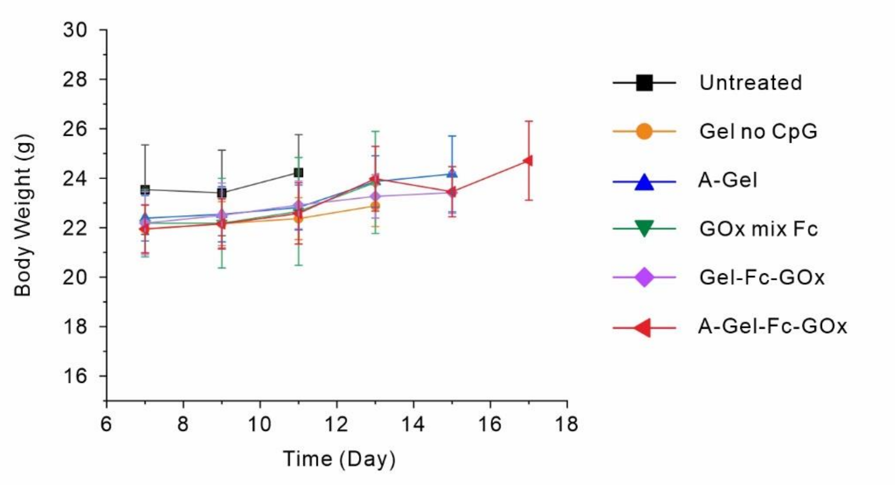

Supporting Figure 7. The Body weight of CT26-tumor-bearing mice with different treatments as indicated.

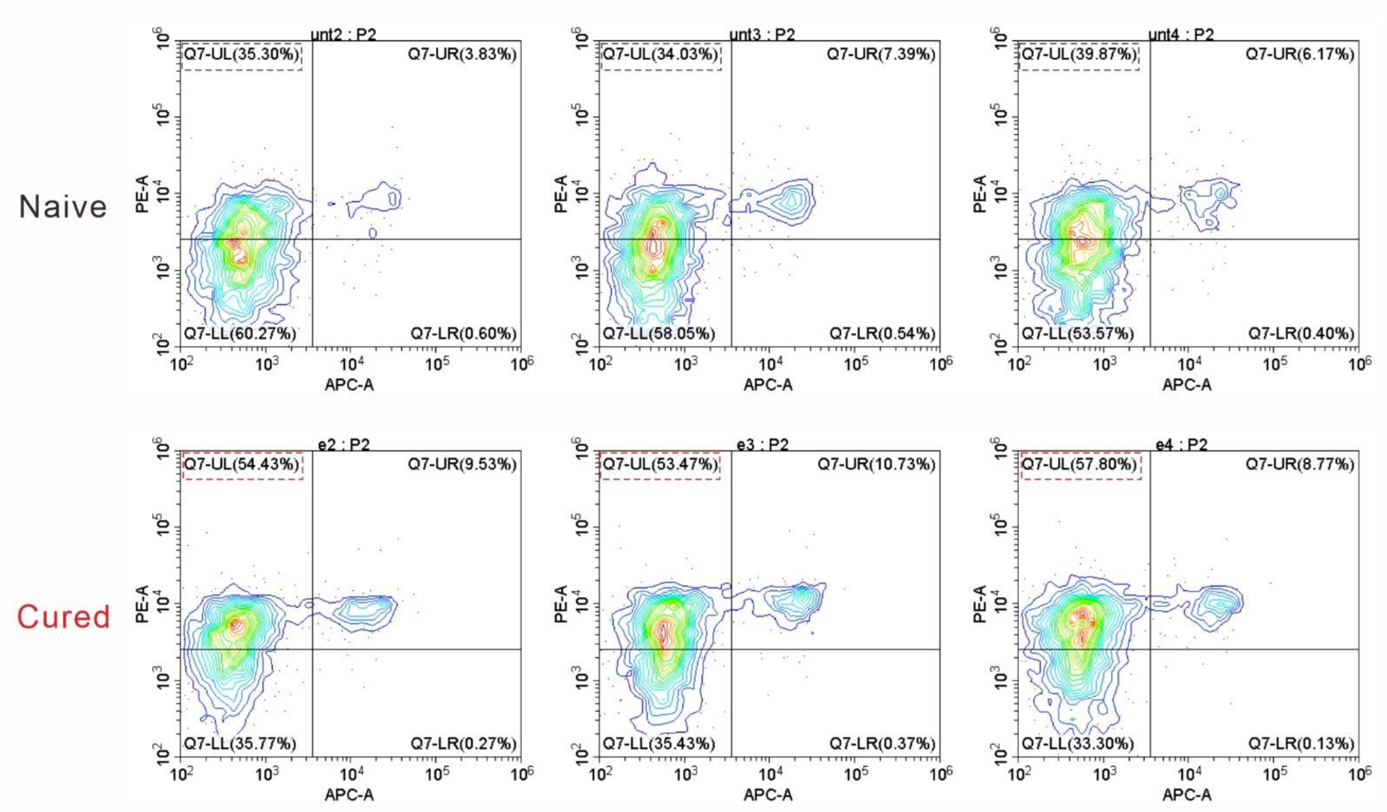

Supporting Figure 8. Represent flow cytometry results showing the percentages of Tem among CD8+ T cells of naive mice and cured mice.

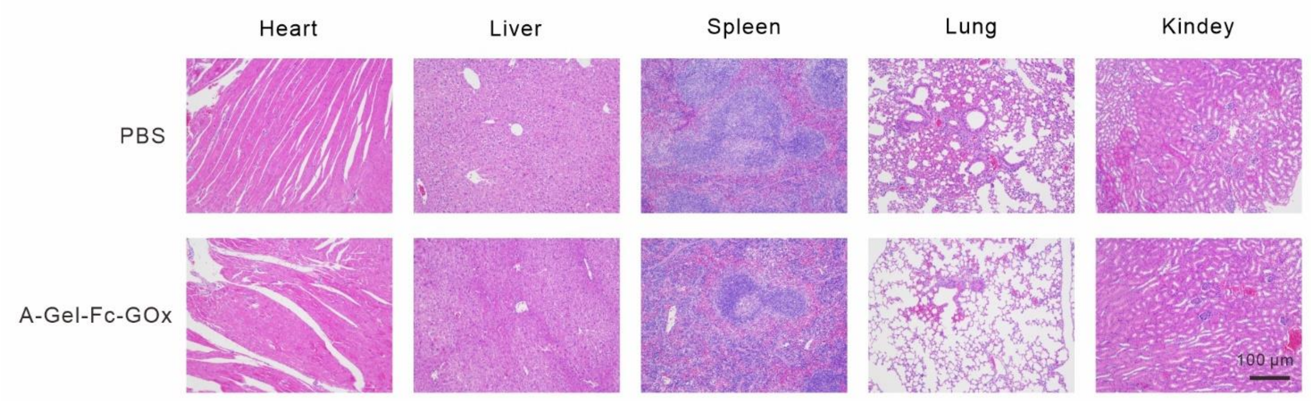

Supporting Figure 9. Histology of indicated organs in mice 72 h after the intravenous injection of PBS and DNA adjuvant hydrogel-Fc-GOx. No observable tissue damage, necrosis, or inflammation was detected. Scale bar: 100 μm.

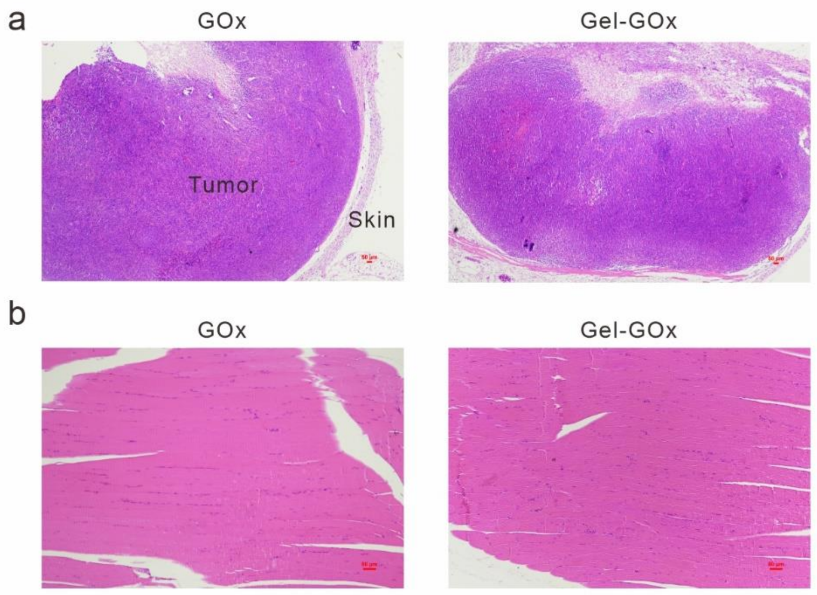

Supporting Figure 10. Histology of local skin (a) and muscle tissues (b) at the injection site. No damage was detected. Scale bar: 100 μm.

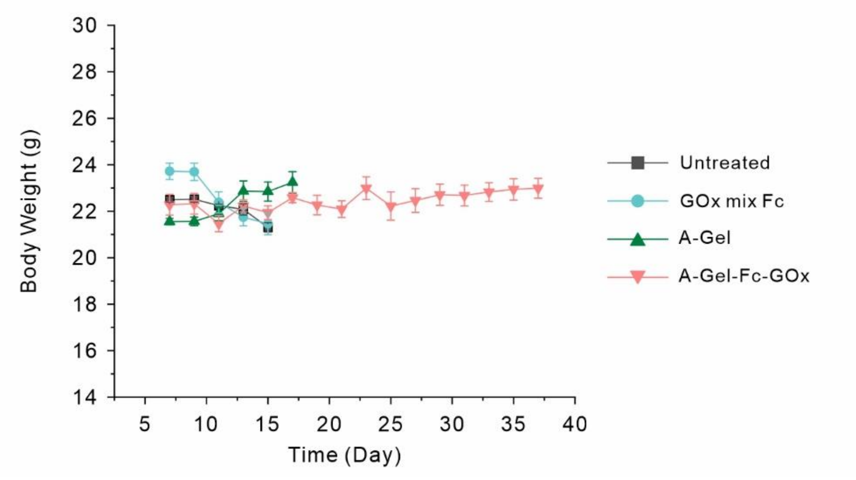

Supporting Figure 11. The Body weight of B16F10-tumor-bearing mice with different treatments as indicated.

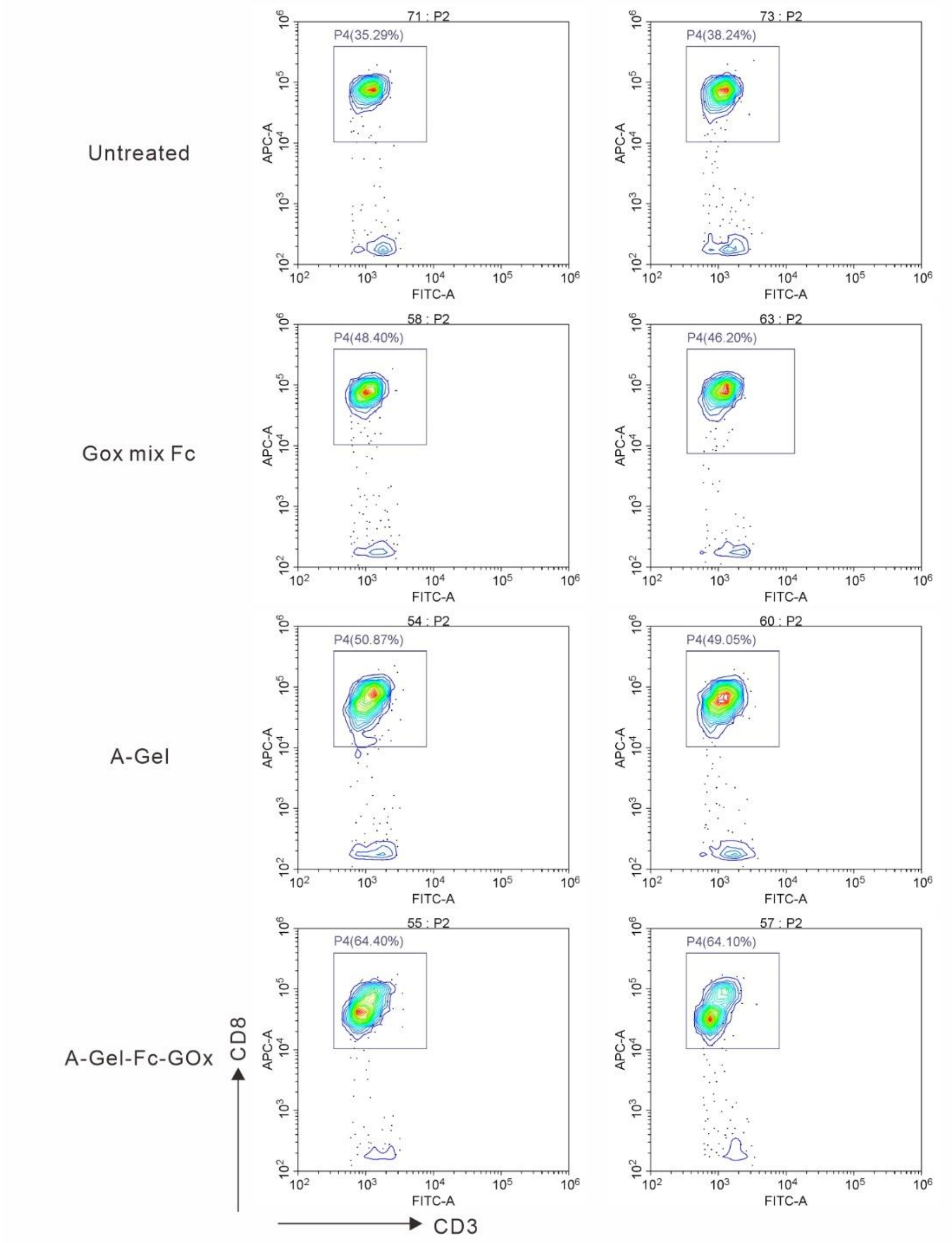

Supporting Figure 12. Flow cytometry results showing the percentages CD8+ T cells maturation levels following different treatments in B16F10-tumor-bearing mice.

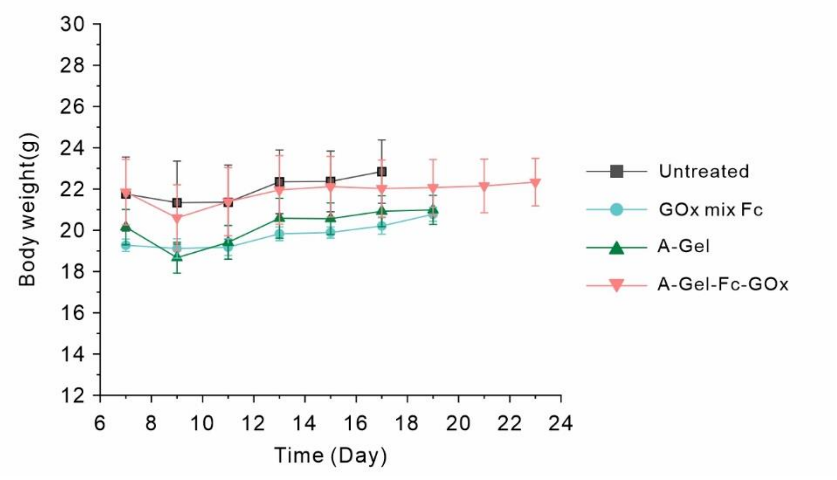

Supporting Figure 13. The Body weight of 4T1-tumor-bearing mice with different treatments as indicated.

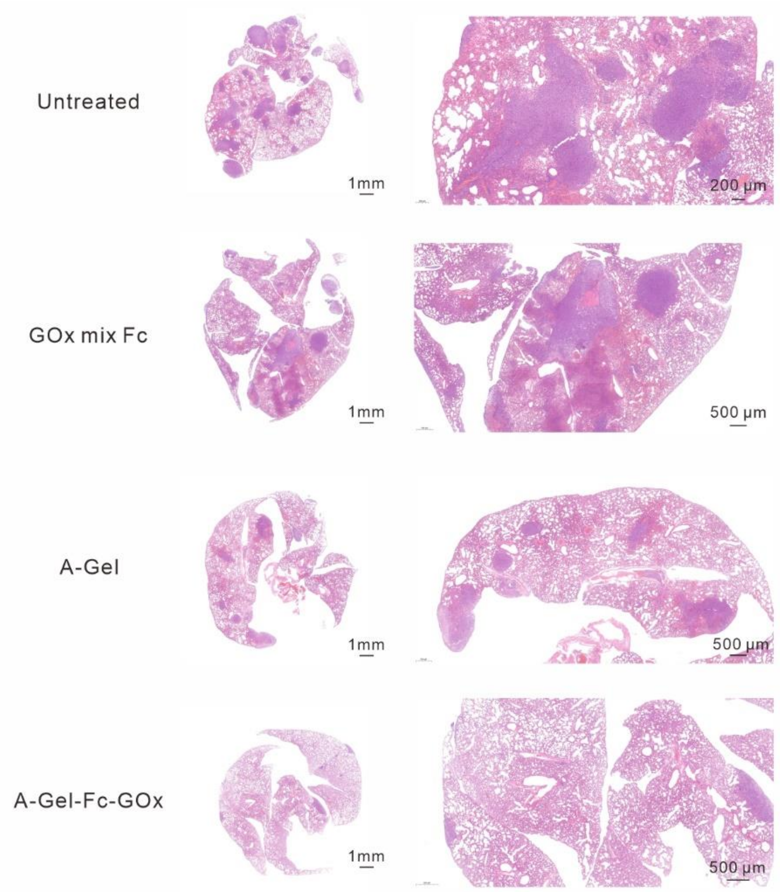

Supporting Figure 14. H&E staining of the lung tissues of the 4T1-tumor-bearing BALB/c mice collected on the 27th day post.
